# Statistical Report on the Sickness, Mortality, and Invaliding among the Troops in the West Indies

**Published:** 1839-01-01

**Authors:** 


### Statistical Report on the Sickness, Mortality, and Invaliding among the Troops in the West Indies. From the Records of the Army Medical Department, &c. Presented to both Houses of Parliament, 1838.

If such records as these had been made and preserved during the tumult, carnage, and destructive sickness of the late war, medical statistics would have been vastly enriched. But the past cannot be recalled; and great credit is due to the Army and Navy Medical Departments for the accuracy and authenticity which they have infused into the reports transmitted to them from all parts of the world. The volume before us is among the first fruits of this energetic system; and the labours of Mr. Henry Marshall, and Captain Tulloch, who compiled this report from upwards of 160 folio volumes of returns, accumulated at the Army Medical Board since 1816, are above all praise. These gentlemen have had a most laborious and difficult task to perform, but the result is a highly interesting and valuable document; especially to all those who are destined to visit the pestiferous but beautiful Isles of the Antilles. From such condensed documentary reports, filled with numerical details, it is no easy matter to draw up an analysis—indeed it is next to impossible; but we shall be able to introduce to our readers a great mass of information respecting the West Indies, which cannot fail to prove interesting even to the practitioner of the remotest village in England.

The Windward and Leeward Islands extend from 6 to 17 degrees of North latitude, and from 56 to 63 degrees of West longitude—stretching in a chain across the great Gulf of Mexico, and differing greatly in climate, aspect, and salubrity. Thus Tobago, Trinidad, St. Lucia, and Dominica, are mountainous, covered with dense forests, and intersected by deep ravines, impervious to the breeze, and where the rains stagnate among a mass of decaying vegetation. On the other hand, Antigua and Barbadoes are comparatively low, barren, and rocky, with dry climate, and agreeable temperature. Some other islands possess a kind of intermediate character, while the coast of British Guiana is totally different from all—being an immense tract of level country, scarcely above the surface of the sea; presenting, during the rainy season, an endless succession of swamps and marshes, with an exceedingly humid atmosphere, though a not very variable temperature. The following leading characteristics of these interesting regions are worthy of note.

"1st. The first peculiarity which distinguishes the climate of this Command is a high temperature, a necessary consequence of proximity to the equator. The mean height of the thermometer throughout the year is, however, rather under than above the average of similar latitudes, being only about 80½°. In none of the islands is it above 82° or under 79°, and any slight difference in this respect results more from their geological features, or extent of cultivation, than the mere difference of latitude; as the mean temperature of British Guiana in latitude 6°, is but 80½°, while that of St. Kitt's, more than 12 degrees further to the North, is 81°.

2d. The next peculiarity which extends to this, as well as most tropical regions, particularly of insular situation, is great uniformity of temperature. The difference between the highest and lowest mean range of the thermometer, is, even in the most variable of the islands, only 13°, and in some it is not more than 4° throughout the year; whereas in Britain it is, in most years, upwards of 30°.

3d. In this, as well as other tropical climates, there is but little change in the elasticity or pressure of the atmosphere. The extreme range of the barometer is not more than from a quarter to half an inch throughout the year, and it is not materially affected even by hurricanes; whereas, in this country, its range is from two to three inches, and it varies with every slight change of weather.

4th. One of the most marked of the atmospherical peculiarities of these regions, is the large quantity of rain which falls annually, being, on the average, at least three times as much as in Britain—a necessary consequence of rapid evaporation under a tropical sun. The quantity, however, varies materially in the different colonies, according as their surface is mountainous or level, clothed in wood, or cleared and under cultivation. We have, in a subsequent portion of this Report, stated the fall of rain in each colony, so far as it has been ascertained by measurement; the average quantity, throughout the whole Command, has been estimated at from 60 to 70 inches annually.

The rain of these regions is, however, of a very different character from that of Britain, being confined principally to two seasons of the year, termed the Spring and Autumnal rains, and then falling not in gentle showers, but in torrents, which, unless in a very dry soil, or where there is free drainage, speedily inundate the surrounding country.

5th. The four seasons of temperate climates are therefore represented by two wet, and two dry seasons; but, as the rains follow the course of the Sun, it is obvious that the period of their commencement and duration must vary according to the proximity of the settlements to the equator. In Guiana, the most southerly, the Spring rains generally extend from December to January, the Autumnal from May to August, while in the most northerly of these settlements, the former does not commence till April or May, and the latter extends from October to December.

In many of the islands, particularly the less hilly ones, there is scarcely any deposition of dew, and in the others it is generally scanty, except in densely wooded districts.

6th. In regions exposed to such a high temperature, it is fortunate that the heat of the day is generally modified by a sea-breeze, not of that variable character which prevails in temperate climates, but which blows with nearly uniform force, and from one direction, during nine months in the year. It is termed the Trade Wind, and generally comes from the East and its collateral points, except from August to December, when it veers round, and blows slightly from the South and West, with frequent calms at intervals.

While this Trade Wind prevails during the day, a land wind, in all the large and mountainous islands, blows with almost equal regularity at night; for as soon as the sea-breeze dies away, the hot and rarified air of the plains, ascending to the mountain tops, is there condensed by the cold, and flows in a steady current towards the ocean, to supply the equilibrium of the atmosphere. In the smaller islands, and those in which there are no mountains, there is either no land wind, or it is very slight. The sea-breeze generally sets in between 10 and 11 A.M., blows with increasing force till 3 P.M., and dies away about sunset, when, after a short interval, the land wind commences, and continues till sunrise.

In these and some other tropical regions, in similar latitudes, hurricanes are occasionally experienced between the month of August and latter end of October, hence denominated the hurricane season. Trinidad, Tobago, and the settlements to the South, have hitherto been exempt from them, but they sometimes fall with dreadful violence on the other islands. Barbadoes, in particular, has suffered very severely from their ravages.

During the rains, particularly at their commencement and termination, thunder and lightning are very common, but seldom occur at any other period of the year. Unfortunately little has been done to investigate the electrical condition of the atmosphere in these parts of the world, and therefore we can only estimate the presence of that agency from its visible effects at these periods." 4.

From several statistical tables inserted here, it appears that, among every 1,000 white troops, there have been annually admitted 1,903 into hospital, so that, on an average, every man must have been under medical treatment, about once in six months and a half. In the United Kingdom the proportion is about one in thirteen months—or just one half. There is a still greater difference in the ratio of severity. In the West Indies there is one death in every 24 admissions, while at home there is only one death in every 67 cases treated. The following table is very curious, and we shall insert it here.

| | Admissions. | | Deaths. | |
|---|---|---|---|---|
| | Total among whole Force in 20 Years. | Annual Ratio per 1000 of Mean Strength. | Total among whole Force in 20 Years. | Annual Ratio per 1000 of Mean Strength. |
| Fevers - - - - - - - | 62,163 | 717 | 3,195 | 36·9 |
| Eruptive Fevers - - - | 13 | $\frac{2}{10}$ | 1 | .. |
| Diseases of the Lungs - - | 9,975 | 115 | 906 | 10·4 |
| ,, of the Liver - - | 1,946 | 22 | 161 | 1·8 |
| ,, of the Stomach and Bowels - | 36,474 | 421 | 1,795 | 20·7 |
| ,, of the Brain - - | 2,447 | 28 | 312 | 3·7 |
| Dropsies - - - - - - - | 659 | $7\frac{8}{10}$ | 180 | 2·1 |
| Rheumatic Affections - - | 4,202 | 49 | 17 | |
| Venereal Affections - - | 5,043 | 35 | 6 | |
| Abscesses and Ulcers - - | 17,708 | 204 | 18 | |
| Wounds and Injuries - - | 11,149 | 129 | 60 | 2·9 |
| Punished - - - - - - - | 4,327 | 50 | 2 | |
| Diseases of the Eyes - - | 7,686 | 89 | 4 | |
| ,, of the Skin - - | 559 | 6 | 1 | |
| All other Diseases - - - | 2,584 | 30 | 145 | |
| Total - - - | 164,935 | 1,903 | 6,803 | 78·5 |

The reader will not fail to remark on the frequency of pulmonary affections, as compared with hepatic diseases or disorders, even in a tropical climate, where the biliary system is supposed to bear the brunt of the malignant influence of such portions of the globe. Affections of the lungs are nearly as 8 to 1 compared with affections of the liver—and the ratio of mortality is nearly the same. Now in the East Indies the proportions would be very different. Hepatic would far outnumber pulmonic complaints. The great proportion of fevers to other diseases is very striking. Sixty-two in every 164! Two-fifths of the fever admissions were intermittents—few of which proved fatal. In the low marshy settlements of Demerara and Berbice, the number attacked in each year being frequently equal to the whole force employed! In Trinidad, too, intermittents are very numerous. Since 1827, only one case of "yellow fever" (febris icterodes) is returned! This results from the arbitrary classification of fevers—many medical officers considering "bilious remittent fever," as a better name than yellow fever.

In respect to the pulmonary affections, though the admissions under this head are less than at home, in the proportion of 115 to 148, yet the mortality is greater—10½ per thousand of whole strength having perished by lung affections. The average per thousand in the United Kingdom was 8½. In the West Indies, 1,023 cases of consumption occurred in an aggregate strength of 86,661—being 12 per thousand annually. In the United Kingdom, the proportion was 5½ per thousand! Inflammation of the lungs and chronic catarrh are twice as prevalent in the West Indies as they are at home—"shewing how little effect a mere increase of temperature has in modifying these diseases."

HEPATIC AFFECTIONS, though by no means so numerous as in the East Indies, were yet nearly three times more frequent than in Europe—and five times more fatal.

GASTRIC AND INTESTINAL AFFECTIONS prove a most fertile source of sickness in the West Indies—being about 421 annually out of every 1000! In England the proportion is only 95 per thousand annually—and very mild—causing only one death in every 2000 of strength. In the West Indies the mortality is 21 per thousand—more than 40 times more than at home! The chief form is chronic dysentery, one-fifth of the cases proving fatal. The greater number of acute dysenteries and diarrhœas prove ultimately chronic, and fatal in the above proportion. Gastritis and enteritis prove also extremely severe in their character, and fatal in their effects.

DISEASES OF THE BRAIN comprehend inflammation, headache, coup de soleil, hydrocephalus, apoplexy, paralysis, epilepsy, fatuity, mania, delirium tremens. At first sight this class would appear to be very prevalent and fatal—the admissions and deaths being nearly four times as much as in Great Britain. More than half of these cerebral affections, however, have resulted from *delirium tremens*, the direct consequence of intemperance, and this last the consequence of the facility of procuring rum.

DROPSIES. The admissions and deaths under this head are very great, in proportion to those at home—being nearly as eight to one. Most of these dropsies are the sequences of fever.

The other classes of disease offer nothing particularly interesting, the mortality being very low. We shall only notice one circumstance. The ratio of venereal affections in the West Indies is 35 per thousand men annually. In Great Britain it is 181—or nearly eight times greater at home than abroad! In the East Indies and Mauritius venereal affections are even more prevalent than at home. This dissimilarity is inexplicable, except by some peculiarity in the climates of the two hemispheres.

BLACK TROOPS. There being no statistical records of the mortality of the

Negroes in Africa, it is difficult to ascertain the influence of a West India climate on them. The average mortality, however, is supposed to be more than in Africa; and, consequently, that the West India climate is unfavourable even to the blacks. It is found, indeed, that black troops, wherever they may be located, as in the Mauritius, Ceylon, &c. present a greater rate of mortality than other troops. The negro corps in Ceylon soon became extinct! The mortality among the negro slave population in the West Indies, is 1 in 33 annually—which, however, is very much less than amongst the negro troops.

### British Guiana.

This comprehends the British settlements on the rivers Essequibo, Demerara, and Berbice. It is in Lat. 6, and Long. 56-60, being about 200 square miles. The soil is deep alluvial, the rich deposits of the foregoing rivers. The country is, of course, as level as the ocean; and but little elevated above its surface. It is principally covered with dense forests and rank grass. In the rainy season much of it is inundated. The climate is, as one would expect, extremely moist. The medium range of the thermometer is 80 degrees, and the land-winds are loaded with vegeto-animal miasmata. The average mortality among the troops, from 1817 to 1836, was 84 out of every thousand annually. In the black corps it was only 41 per mille. Bowel-complaints are not so prevalent here as in most other West India stations; but diseases of the brain, especially delirium tremens, were enormous—owing to rum.

### Trinidad.

This island is separated from the great American continent by a narrow strait, only 12 miles across. It is about 70 miles long by 50 broad. Its mountains rise to 3,000 feet above the sea, broken into the most rugged masses, and clothed to their summits with majestic forests. The island is well watered; but the greater part of the interior is uncultivated. The nights are cool and pleasant; but the daily temperature is about 80°.

Among the white troops the ratio of annual mortality has been 106 per thousand. This high rate has been chiefly caused by fevers and dropsies. A severe epidemic fever in 1818, cut off nearly a third of the force on the island.

### Tobago.

This island lies close to Trinidad, and appears from the sea like a mass of dark abrupt precipices. The climate and seasons are much the same here as in Trinidad, but the atmosphere is much more humid. The soldiers have been, in general, very unhealthy in this island. Thus, on the average of 20 years—1817 to 1837, the mortality has amounted to about 153 per thousand of white, and 34 per thousand of black troops *annually!* This is nearly double the mortality of the Windward Islands generally, as well as the Leeward. The great cause of mortality is fever—next, gastro-intestinal affections—and thirdly, pulmonic complaints.

### GRENADA,

Is a small island, with mountains of 3,000 feet. It is more cultivated than most islands of a mountainous character—the soil of the vallies is very rich. The average annual mortality among the white troops, has been, for 20 years past, 61 per thousand. The climate of this island is, therefore, more favourable to life than the general average of the other islands in the West Indies. The deaths annually were, by fevers, 26—by gastro-intestinal affections, 16—by pulmonary affections 6 per thousand.

### ST. VINCENT'S.

This is a small island, 18 by 11 miles—the central mountains 4,000 feet in height. There is a considerable space of level ground on the coast, affording rich crops. The island is of volcanic origin, and a violent eruption took place in 1812. The mountains are clothed with immense forest trees, with very little jungle or underwood. The ventilation is not therefore impeded. The vallies are wide, and free from excessive vegetation, presenting a character of salubrity to the experienced eye. There is very little swamp on this island, but it is well watered by numerous rivulets. The atmosphere is humid, and rain is common during most part of the year—the average quantity that falls being from 70 to 80 inches. The dews are very heavy. The heat is nearly the same as in the other islands. The ratio of mortality has been found to be 54 per thousand per annum. The climate is, on the whole, more salutary than the usual run of the West Indies. In the list of maladies, the gastro-intestinal affections are more than double of the fevers, being as 180 to 83. This is very remarkable.

### BARBADOES.

This island is 22 by 16 miles in extent. It rises from the level of the sea by a succession of terraces to the height only of 1,100 feet. It presents a much more barren and inhospitable aspect than the other West India islands from the sea. The soil is light, calcareous, and absorbent. The fall of rain is only 58 inches annually, and the dews are light. The thermometer ranges the usual height, but the heat is not at all oppressive. The trade wind, from the South-east, blows both day and night, but very feebly after sun-set. In the first four or five months of the year, it is strong and regular; and the climate is healthy and pleasant. June, July, and August are hot and disagreeable—and, in the latter month, the hurricane season commences; but fortunately is not of frequent occurrence. The average mortality among the white troops, during the last 20 years, has been 58 per thousand per annum. That of the blacks was 46 per mille. Diseases of stomach and bowels were to fevers as 498 to 282. The lungs were as 379 to the above numbers. Liver 34—brain 80—dropsies 58.

" From this it appears that the principal causes of mortality among the white troops have been diseases of the lungs and of the bowels. The deaths by the former are considerably above the average of what prevails throughout the whole

Windward and Leeward Command; but it must be kept in view that, as Barbadoes is the station to which invalids are generally sent, for the purpose of obtaining a passage to England, many of those labouring under consumption, contracted in the other islands, have died there, and, no doubt, increased the mortality by diseases of the lungs much beyond what could properly be attributed to the climate of that island. Diseases of the bowels constitute more than a third part of the whole deaths, and seem not to be diminishing of late years either in frequency or severity. They proved most fatal in 1817, when dysentery was extremely prevalent, particularly in the 2nd, or Queen's Regiment; so much so that about the middle of the year, nearly half of that corps were in hospital from it. Various means were adopted to protect them against the influence of this disease, but without effect." 28.

### St. Lucia.*

This island is 32 miles in length, by 12 in breadth, and lies about 40 miles to the north of St. Vincent's. Basseterre, the lowest and best cultivated portion of the island, abounds in swamps and marshes. Capesterre, on the contrary, consists of abrupt and picturesque mountain scenery, resplendent with woods, and intersected by numerous ravines, too narrow to admit ventilation, and replete with moisture and miasmata. Rains are frequent for nine months of the year; during the other three the weather is pleasant. The ratio of mortality among the troops during the last 20 years is as follows:—Whites 122 per mille per annum! Blacks 42 ditto. Ratio of diseases, fevers 304—gastro-intestinal complaints 189—pulmonary 60—brain 21—liver 5—dropsies 9. All other diseases 3.

From this it appears, that fevers and bowel-complaints produce terrific havoc in this island. St. Lucia, indeed, has always been noted for the insalubrity of its climate.

### Dominica.

This island is 29 miles by 16, and lies midway between the French islands of Martinique and Guadaloupe. It very much resembles St. Lucia in its physical characters. The mountains rise to 5,000 feet in some places. It is of volcanic origin. The mortality has been 137 per mille per annum among the white troops, and 39 among the blacks. Gastro-intestinal affections 332—fevers 233—pulmonary 39—hepatic 8—brain 25—dropsies 3—all other complaints 9. Thus more die of bowel-complaints than of all the other diseases put together!

---

* We are going more into details than English readers may think necessary. But these statistics are of exceeding value; and as medical practitioners are often consulted in this country respecting the climate of the West Indies, it is politic that they should possess a little more than the vague notion that these islands are, as Jonathan would say, "tarnation hot and unhealthy." Pulmonary invalids very often go now to Barbadoes, Jamaica, and other islands, under the idea that consumption cannot exist within the tropics. These facts will give them a little clairvoyance on the subject.—*Rev.*

### ANTIGUA AND MONTSERRATT.

The latter island is only a dependency of the former, and distant from it about 22 miles. Antigua is comparatively flat, the greatest elevation being 1,210 feet. Much of the surface is barren—some of it swampy—and the climate is characterized by dryness—the average fall of rain not being more than 45 inches. Montserratt is more mountainous, having, in some places, an elevation of 2,500 feet. This island is noted for its salubrity. The average mortality in these two islands was 40 per mille per annum among the whites, and 28 ditto among the blacks. The mortality among the whites is not more than half the average mortality of the West Indies generally. The proportions were:—fevers 120—pulmonary 73—gastro-intestinal 74—liver 23—brain 15—dropsies 11. All others 11.

It will be seen from this, that though the mortality by all complaints put together is low, the pulmonary scourge maintains its ground.

The islands of St. Christophers, Nevis, and Tortola, present nothing remarkable. The ratio of mortality amongst the white troops was 71 *per mille per annum*. Fevers 244—stomach and bowels 60—pulmonary 55—liver 13—brain 16—dropsies 5. All others 19.

### JAMAICA.

This island lies 900 miles west of the Islands or Command which we have noticed. It is 170 miles in length by 50 in breadth. The range of mountains throughout its length rises in some places to 8,000 feet—nearly as high as the Great St. Bernard amongst the Alps. This range being nearly east and west, forms a complete barrier between the north and south sides of the island. On approaching from the south, the immense mass of blue mountains bursts on the view—their lower range crowned with wood, and sloping down to a plain of ten or twelve miles in breadth along the coast, on which the principal towns are built. The soil is red and rich, and watered by innumerable streams. On the north side, there are few plains, or level surfaces of any extent: the ground rises from the sea by a succession of acclivities, separated by wide valleys, the hills being conical, and studded with thick groves of the pimento tree. These features gradually blend into the alpine scene aloft, covered with forests of cedar.

"The interior of the island has quite a different appearance from either side, presenting all the varieties of feature peculiar to a highland district; in some parts rugged, difficult of access, and densely wooded; in others spreading out into a wide expanse of table land or elevated plain, from which rise a number of small hummocks, giving a slight undulation to the surface. The ground in such spots is generally clear and open, covered with rich grass, and of a pastoral character. These regions, in which cultivation has made but little progress, are principally used for the rearing of cattle.

As these portions of the island present varieties in physical aspect, so they exhibit a corresponding diversity in climate. On the plains or sea-coast of the south side, the thermometer at noon does not vary more than 8° or 9° throughout the year, its greatest height being about 92°, and lowest 83°. The mid-day heat on both sides of the island is greatly modified by the influence of the seabreeze, which generally sets in from the eastward about eight or ten o'clock in the morning, increases in force till about two, and declines with the sun, till, on the approach of evening, it is succeeded by the land wind from the mountains. When these winds become less regular, or altogether fail, as is sometimes the case before the rainy season, the atmosphere is exceedingly oppressive to the feelings, though the thermometer perhaps exhibits but little change in the temperature.

The quantity of rain which falls throughout the year is about 50 inches, and the seasons may be distinguished as follows. From the middle of December to the middle of April there is generally clear dry weather, except a few showers at Christmas. During the first three months of this period, north winds prevail, with considerable diminution of temperature, the thermometer sometimes sinking so low as 70° in the morning, but by mid-day it generally stands at from 83° to 85°; the sea-breezes at this time are weak and irregular. About the middle of April the sea-breeze fails altogether, the thermometer rises to about 86° at mid-day, and is seldom below 80° at night; the heat becomes oppressive, the atmosphere cloudy, and a few transient showers begin to usher in the Spring rains, which continue with great violence during most part of May, and are generally preceded by heavy storms of thunder and lightning. The weather in June is generally hot and dry, the sky is seldom obscured by a cloud, the thermometer often rises to 92° at noon, the land-wind fails, and the nights are consequently oppressive, but the sea-breeze is strong, and tends greatly to moderate the intensity of the heat. Very little change is perceptible throughout July or August, but in September there is the same close sultry weather as in April, which continues till the Autumnal rains set in, about the middle of October. These are generally preceded by thunder and lightning, though not to so great an extent as in Spring, and continue from four to six weeks, during which period the temperature undergoes a reduction of about three or four degrees by day, and six or seven by night.

From July to October are the hurricane months in this island, but fortunately these phenomena are of rare occurrence.

The seasons on the north side of the island are somewhat different, the rains being generally a month later in their commencement, and much longer in their duration than on the south side; a greater quantity also falls, and the showers are more equally distributed throughout the year. Owing to the vicinity of the mountains, and there being little extent of level ground, the atmosphere is cooler in Winter, and more liable to sudden alternations of temperature.

The high lands in the interior of the island possess a very different climate from that of either side, their great elevation producing a corresponding diminution of temperature, which would be still more perceptible, were it not that the sea-breeze, which modifies the heat of mid-day in the low country, does not extend to the mountains, and is even but partially felt at the distance of a few miles from the coast. It is, consequently, in the morning and evening that the diminution of temperature is most felt in the high grounds; at which periods it sometimes exceeds 25°." 43.

In this island almost every variety of climate may be obtained. At a height of 4000 feet, the thermometer stands at 55° to 65°—in Winter it falls to 44°. There the tropical vegetation ceases, and is supplanted by that of temperate regions. Showers are common in the interior of the island throughout the greater part of the year. The air is very humid, and dense fogs are frequent. While the yellow fever is desolating the plains on the coast, these mountains enjoy a complete immunity from the pestilence. It has never been known to extend above an elevation of 2500 feet—a proof, if proof were wanting, that it is not propagated by contagion. The inhabitants of these regions enjoy health and long life, and present ruddy complexions like those extra-tropical countries. The ratio of mortality, for 20 years, has been as follows:—121 annual deaths out of every 1000 of strength. But the real mortality, owing to some omissions in the returns, &c. was 143 *per mille*.—A tremendous wear and tear of human life! In the most healthy periods of the last twenty years, the mortality has been four or five times as much as among troops at home—in the sickly seasons sixteen times as much! It was found that the average mortality in Jamaica, for the thirteen years preceding 1817, was nearly as possible the same as since: so that the climate has experienced no amelioration—nor have the means of preventing or curing diseases increased in efficiency.

In the twenty years past, fevers occasioned 101 deaths *per mille per annum*—lung diseases, 7 ditto ditto—liver, 1 ditto ditto—stomach and bowels, 5 ditto ditto—so that pulmonic diseases stood first after fevers in mortality. Jamaica is much more infested with the fatal bilious remittent fever than the Windward and Leeward Islands.

"Several instances will be adduced in the course of this Report to show that these epidemics spared neither age, sex, nor condition of life; the temperate and the intemperate, the prudent and the thoughtless, fell victims to them in nearly an equal degree; and all sanatory precautions, save the immediate removal of the troops from the locality where they originated, seem to have had little or no effect in arresting their progress. Their appearance cannot be said to have been confined to any particular season of the year, for the results on this head are by no means uniform, though the preponderance of mortality has been greater during the last two than the first two quarters, as will appear from the total deaths stated in each Quarterly Report, during the years when they were most prevalent and fatal in the island." 46.

In this place the reporters observe that, "unlike the yellow fever of Gibraltar, one attack of the remittent fever of this country secures no immunity from a second." Here the reporters have deviated from the sober path of facts, to the seductive walk of theory. What business have they with the "yellow fever of Gibraltar?" Even if they had been agents in the Gibraltar fever under the Frasers, the Pyms, and contagionists in general, they had no right to lug it in here, without anything on the record to bear them out.* Such a procedure in a Court of Law or Justice would be instantly quashed. But it discloses to us a bias, a *prejudice*, which casts a suspicion on every conclusion to which the reporters come on the data before them. They have thus, to use a vulgar expression, "let the cat out of the bag," a great deal too soon for themselves, and very fortunately for their readers.

But to return to facts. Of the pulmonary affections admitted into hospital, in Jamaica, the following was the ratio of mortality.

* The proofs or rather the assertions that one attack of the Gibraltar fever will secure from subsequent attacks are almost entirely *ex parte*.

" DISEASES OF THE LUNGS.

Under this head are comprised in the preceding Table—

| | Admitted. | Died. | Proportion of Deaths to Admissions. |
|---|---|---|---|
| Inflammation of the Lungs ........ | 697 | 15 | 1 in 46 |
| Pleurisy ........................ | 29 | 0 | 0 in 29 |
| Spitting of Blood ................ | 108 | 12 | 1 in 9 |
| Consumption .................. | 661 | 315 | 1 in 2 |
| Acute Catarrh.................... | 2,438 | 18 | 1 in 135 |
| Chronic Catarrh.................. | 371 | 23 | 1 in 16 |
| Asthma ........................ | 41 | 3 | 1 in 14 |
| Difficulty of Breathing ............ | 12 | 2 | 1 in 6 |
| Total.... | 4,357 | 388 | 1 in 11 |
| Annual ratio per 1000 of Mean Strength .................... | 85 | 7·5 | ...... |

This class of diseases has been about one-third less prevalent, and one-third less fatal, than on the Windward and Leeward Commands. But when the deaths on the voyage home, and after landing in England, are taken into account, it appears that the wear and pulmonary affections in the West Indies, is, as nearly as possible, the same as in an equal number of troops in Great Britain.

" As an instance how much more prevalent consumption is in that country than in Britain, we may state that of an aggregate strength of 51,567, serving in Jamaica, there have been 661 treated for that disease, being at the rate of 13 per thousand annually ; while out of an aggregate strength of 44,611 dragoon guards and dragoons, serving in the United Kingdom, there have been treated only 286, or between 5 and 6 per thousand annually, and that, too, though the period over which the latter observations extend, includes two severe epidemics of influenza, which, no doubt, laid the foundation of more cases of this disease than usually occur in this country."

" The baneful influence of the climate of the West Indies in accelerating the progress of consumption has been often remarked by the medical authorities; but it does not seem to have occurred to them, nor indeed had they any means of ascertaining, that at least twice as many cases of it originate in that climate as at home, though those catarrhal affections to which they are generally attributed are there comparatively so rare." 47.

This, we think, may open the eyes of those who consider a hot climate a panacea for pulmonary complaints.

DISEASES OF THE STOMACH AND BOWELS.

The following table shews the proportions which the various kinds of gastro-intestinal affections bear to one another.

| | Admitted. | Died. | Proportion of Deaths to Admissions. |
|---|---|---|---|
| Abdominal Inflammation .......... | 2 | 1 | 1 in 2 |
| Inflammation of the Stomach ....... | 42 | 4 | 1 in 10 |
| Ditto of the Bowels.............. | 52 | 11 | 1 in 5 |
| Vomiting of Blood ............... | 9 | 4 | 1 in 2 |
| Acute Dysentery. ............ .. . | 4,473 | 114 | 1 in 40 |
| Chronic Dysentery................ | 436 | 70 | 1 in 6 |
| Indigestion ..................... | 579 | 5 | 1 in 116 |
| Colic ........................... | 1,107 | 4 | 1 in 277 |
| Obstipation ..................... | 196 | 2 | 1 in 98 |
| Cholera Morbus .................. | 216 | 3 | 1 in 72 |
| Diarrhœa..... .................. | 5,169 | 42 | 1 in 123 |
| Cancer of the Stomach ........... | 1 | .... | 0 in 1 |
| Total.... | 12,282 | 260 | 1 in 47 |
| Annual ratio per 1000 of Mean Strength .................... | 238 | 5·1 | |

Under the head of diseases of the brain, we were surprized to see so large a number as 258 cases of epilepsy out of 720 admissions. The mortality, too, was 1 in 14! Rum, however, is the cause of all! Venereal diseases are veiy rare in Jamaica.

We must pass over descriptions and tables of various localities in Jamaica, with the exception of a curious place—MARVON-TOWN, situated among the mountains, and elevated 2000 feet above the level of the sea. It is about 18 miles from Montego Bay, and commands a magnificent prospect by sea and land. Being surrounded by high mountains, the climate is very variable, and the atmospheric vicissitudes considerable. Much rain falls, and the evaporation by a tropical sun is great, producing dense fogs. The thermometer seldom rises above 80°, and is often, at night or towards morning, so low as 52°. The actual mortality at this station is not above $1\frac{1}{2}$ per cent. per annum, which is, as nearly as possible, the mortality among the Foot-guards in London. There are several other mountain-stations, as Greencastle, Phœnix Park, Montpellier, Mandeville, &c., where salubrity is the order of the day, and yellow fever never shews its face.

We must pass over the Bahama Islands, a crescent of 600 miles, and containing several hundred isles, chiefly Coraline, and the great majority uninhabited. New Providence is the principal station for troops, and very inimical it is to human life.

Honduras is nearly on a par, in respect to mortality, with the Windward and Leeward Islands.

There is a curious section on the influence of age and length of residence on the mortality of troops serving in the West Indies, from which it appears that, instead of the mortality decreasing with the advance of age, as has been generally supposed, it increases, with infinitely greater rapidity, than in Great Britain. And this has been found to obtain in every station, whether temperate or tropical. Thus the doctrine of seasoning or acclimatization falls to the ground before statistical facts.

In another section a great number of tables and data are brought forward to prove that the mortality among officers, as compared with the men, is about one half. The officers suffer less by fever and rather more by hepatic affections than the private soldier.

The sixth and last section contains deductions from the preceding reports and data.

The reporters observe, that although the minute trains of investigation into which they have gone have not enabled them to "distinguish with certainty the essential causes of sickness and mortality among European troops and civil residents in the West Indies, yet the numerical results at which they have arrived seem sufficient to warrant the belief that many of the opinions hitherto entertained, in regard to the nature and influence of these causes, must have been adopted on very inadequate evidence." Thus the high temperature of the West Indies, they say, cannot be the cause of fevers, since the mean temperatures throughout the whole of the islands is nearly the same, while fevers are ten times more prevalent in some islands than in others. But who, we ask, ascribed fevers to mere temperature? In the East Indies the temperature is much higher than in the West, yet fevers are much less prevalent. But solar heat, though it may not, *per se*, occasion fevers, it may be, and we believe is very instrumental in the production of these and other diseases, not only by increasing the susceptibility of the human frame, but in calling forth those exhalations or miasmata, which occasion such havoc among Europeans in hot countries. It is now nearly thirty years since Dr. Johnson pointed out that it was to *atmospherical vicissitudes, and terrestrial emanations* most of our tropical diseases were attributable—not merely to high temperature *alone*, excepting in as far as it predisposed the body to be noxiously impressed by cold and miasmata.

The reporters go on to "excessive moisture," as a supposed cause of fevers, &c. They observe that, if this were the essential cause of fevers, we ought to have most of these diseases where most rain falls, which is not the case. Certainly not. If mere moisture caused fevers, those who lived on the ocean would never be free from them. But moisture has an injurious effect on the human frame when conjoined with vicissitudes of temperature—but, above all, the evaporation of moisture, in certain malarious localities, carries up those deadly miasmata from the earth which are so destructive of life. The reporters, indeed, acknowledge the insalubrity of heat and moisture *conjoined*.

"Though heat and moisture are not the primary causes of fever, however, it is highly probable their operation tends in some measure to increase its intensity. The Tables, illustrating the influence of the seasons on the health of the troops in each station, show that the greatest number of admissions into hospital and deaths has, on the average of a series of years (though not uniformly or equally in each year), taken place in those months when the greatest degree of heat was combined with the greatest moisture; and it may be observed as a striking exemplification of this fact, that as the sun proceeds northward in the ecliptic, carrying heat and moisture in his train, the period generally termed the unhealthy season, is later in the northern colonies than in those to the south." 102.

The reporters very properly disclaim the idea of miasmata being wafted by the winds from the vast forests and savanhas of South America by the S. W. winds. The reporters attempt to upset the hypothesis, as they term it, of the extrication of malaria from the soil, by very unsatisfactory arguments. Thus, because the physical characters of two localities are apparently the same, and yet they are not equally healthy, miasmata can have nothing to do with the production of fevers!!! With such reasoning it is useless to reason.

So again, that marshes, swamps, and lagoons cannot harbour or emit febrile miasmata, is, they think, proved by the fact that, in very marshy places, as Guiana, Honduras, &c. fevers are not so frequent as in some places in Jamaica, where marshes are little observed. The medical world are well aware that malaria may issue from localities where no marshes exist, as in Rome, for example; but the almost universal fact that swamps and marshes are unhealthy in hot countries, is a sufficient proof (however numerous the exceptions of the other kind) of the connexion of febrific miasmata with fevers and morasses. We know little or nothing of the nature of miasmata that issue from the Pontine marshes, or the vicinity of Rome, no more than of those which emanate from certain localities in the West Indies and other countries; but there are hardly any facts in medical history so well established as the emanations of malaria from the soil of those countries. It is vain to say that the marshes always exist, whereas the fevers are much more violent one year than another; and that in some years they hardly occur at all. We may deny the existence of variolous contagion in the air, because in some years it is ten times more destructive than in others. The reporters would seem to have a great inclination to doubt the security which elevation is supposed to confer on people in hot climates, but the facts which they have already stated, render scepticism on that point impossible.

"The instances of Fort St. George at Tobago, Morne Fortuné at St. Lucia, and Morne Bruce at Dominica, demonstrate that mere elevation to the height of 600 or 700 feet, instead of securing a healthy position, seems rather to have the reverse tendency." 103.

The topographical researches of medical men, in all quarters of the globe, have long proved that miasmata will rise to a certain elevation, and be carried by currents of air to a certain distance; but the exemption from malaria at a height of some thousands of feet, shews that there is a limit to the range. At a considerable elevation (3000 feet or so) grounds capable of emitting malaria may exist, but the low temperature of the mountainous heights checks the extrication effectually.

The reporters, after having, as they supposed, demolished the theories of preceding writers, seem a little inclined to start an hypothesis of their own; but they are too diffident on this point, and merely throw out a hint for others to improve upon.

"We are too sensible of the difficulty of the subject to venture on any theory of our own, which might on subsequent examination prove as futile as those which preceded it; but we merely wish to call the attention of such persons as may be disposed for further inquiry, to the circumstance that as yet no experiments have been made on the electrical condition of the atmosphere in the West Indies, during periods of epidemic; and as it is possible either an excess or deficiency of that powerful though unseen agent, may exercise an important influence on the vital functions, the subject seems worthy of attention. Heat and moisture are well known to be intimately connected with the development of electrical phenomena, and its influence on vegetation has also recently been established by experiment; consequently, if the prevalence of disease could be satisfactorily traced to that source, the reason why heat, moisture, and vegetation should have been mistaken as the causes, when acting only as auxiliaries, would be readily accounted for; and even should the results leave the cause of disease as undetermined as before, science will at least be benefitted by the inquiry." 103.

Passing over the electrical hypothesis (which is not new) we beg to say that, while we differ widely from the reporters in respect to their conclusions —"in which nothing is concluded"—we tender them our unfeigned thanks for the valuable mass of facts which they have accumulated in this volume. These facts will stand when the deductions drawn from them are forgotten— and will furnish data for future speculators, when the present race is no more. We hope the editors will be spared their health for the arduous labours in which they are engaged, and we shall hail with pleasure the advent of another report from the same hands.

P. S. Could they not give us some pathological and therapeutical details, as well as statistical?

